# *Law n. 22,537/2024* of the State of Goiás, Brazil, on awareness against abortion: a critical analysis

**DOI:** 10.1590/0102-311XEN016724

**Published:** 2024-06-21

**Authors:** Roberta Siqueira Mocaiber Dieguez, Cristiane da Silva Cabral

**Affiliations:** 1 Faculdade de Saúde Pública, Universidade de São Paulo, São Paulo, Brasil.

The Legislative Assembly of Goiás, Brazil, sanctioned *Law n. 22,537/2024* ^1^ on January 11, 2024, which institutes the “Awareness Campaign Against Abortion for Women in the state of Goiás”. Proposed by then-state deputy Fred Rodrigues, it provides for measures to combat abortion in the state of Goiás. However, the document makes no mention of the legal allowances for abortion in Brazil: (i) risk to the life of the pregnant woman; (ii) pregnancy resulting from rape; and (iii) fetal anencephaly. Although the target of a Direct Action of Unconstitutionality (ADI 7,597) in the Brazilian Supreme Court, the law remains in force.

In line with the main strategies recently adopted by groups against abortion ^2^ and other Bills that are being processed or have recently been processed in the Brazilian National Congress ^3^ ^,^ ^4^ ^,^ ^5^, the central argument of the Goiás’ document revolves around protecting the life of the “unborn”. Attributing subjectivity to the unborn fetus aims to produce a false conflict of rights between the pregnant woman and her fetus ^6^, clearly violating the reproductive rights of pregnant persons.

In paragraph 1 of Art. 3, the document provides for the development of lectures aimed at children and adolescents on the supposed “risks” of abortion, without distinguishing between clandestine (which are largely unsafe) and legal abortions. The recent scientific literature warns of the risks of unsafe abortion, especially in countries with more restrictive laws, such as Brazil ^7^. The efficacy and safety of legal abortion ^8^ is ensured under the most recommended methods ^9^.

However, this proposal draws attention by focusing on children and adolescents, who are the main victims of sexual violence in Brazil. The most affected by this problem are girls (88.7%) aged from 10 to 13 years (33.2%) ^10^. The law approved in Goiás intends to misinform children and adolescents who are possibly victims of sexual assault and, therefore, have the right to legal abortion. It is worth mentioning that pregnancy in children aged under 14 years configures statutory rape. Therefore, the pregnant woman has had the right to abortion provided by law since 1940.

Another guideline, in paragraph 2 of Art. 3, provides for information to the population about contraceptive methods to prevent unplanned pregnancies. While expanding access to information about contraceptive methods is a positive action based on a public health perspective, including this topic in an anti-abortion law suggests that unwanted pregnancies primarily stem from a lack of access to information. However, the effective use of contraception goes beyond access to knowledge on the subject as it involves a complex network of social relations, such as social markers of difference (social class, race, gender, structuring of services, etc.), inequalities in affective-sexual relationships, and individual history ^11^. Thus, the immediate association between contraception and lack of information reinforces a narrative that blames only women for pregnancy, exempts men, and disregards the complexities of contraception. The aforementioned law includes yet another mistake, reinforcing the stigma of unforeseen pregnancy as women’s failure to regulate their fertility ^12^.

The topic with the greatest repercussion of the approval of the text was the “compulsory provision” of ultrasound for pregnant women, who are now obliged to listen to the heartbeat of the fetus (Art. 3, § 6). In addition to constituting obstetric institutional violence, this measure aims to precisely curb the intention of those who refuse to continue the pregnancy. As observed in another study ^13^, the use of ultrasound in abortion care confers “attributes of a social subject” to the fetus, favoring the moral condemnation of the woman who chooses abortion and putting her at risk of being denounced.

Finally, the text proposes “*to stimulate the private sector and NGOs in the promotion of means to welcome, guide, and provide psychological and social assistance to pregnant women who wish to have an abortion, always prioritizing the maintenance of the life of the unborn child*” (Art. 3, § 5). The prioritization of the life of the fetus prevails over the pregnant woman’s right to legal abortion, which induces the maintenance of an unwanted pregnancy, even if the right to interruption is protected by law. Thus, it configures a way of producing moral, psychological, and institutional impediments for those who need to interrupt their pregnancy, increasing the immense number of current barriers for access to abortion provided for by law.

Barriers and difficulties in accessing safe abortion can damage the health and living conditions of the involved persons. Maintaining pregnancies by refusing abortions can lead to psychological, social, economic, and relational consequences, among other outcomes ^14^. In health, the denial of abortion can lead to higher risks of conditions such as eclampsia and postpartum hemorrhage, and may even increase the risk of maternal death ^15^, in addition to being associated with higher rates of postpartum depression ^16^. Moreover, proposals that restrict access to legal and safe abortion contradict the most recent recommendations of the World Health Organization ^8^, which considers that less restrictive abortion laws are an important step toward promoting sexual and reproductive health and rights of women and men.

Therefore, the approval of *Law n. 22,537/2024* aims to prevent access to legal abortion and to impose pregnancy in cases of rape, violating the Brazilian Penal Code, which confers the right to abortion in these cases. Although state laws are unable to change federal law, the proposal imposes unnecessary psychological suffering on pregnant women by forcing the maintenance of pregnancies resulting from rape and worsens the existing barriers to access related to medical conscientious objection, gestational time limit, service distribution ^17^ ^,^ ^18^ ^,^ ^19^, among others.

Although the change will only directly impact the state of Goiás, its approval contributes to strengthening similar bills in other states of Brazil. These changes in legislation are strong institutional mechanisms that interfere with reproductive autonomy and management (not only of people who become pregnant), and contribute to producing moral regimes ^20^ ^,^ ^21^ ^,^ ^22^ that act coercively on subjects, defining their ways of living. Currently, several bills in Brazil are in line with a neoconservative and anti-abortion agenda, proposed by representatives of the municipal, state, or federal executive branch. Added to this are other recent attacks on sexual and reproductive rights, which manifest themselves, for example, in the closure of referral services for legal abortion care, such as the Vila Nova Cachoeirinha Hospital, in the municipality of São Paulo. Considered one of the only hospitals to offer the procedure after 22 weeks of gestation, the mayor of the municipality ordered the interruption of the legal abortion service in December 2023, alleging that it prioritizes other procedures and surgeries ^23^. There followed the dismissal of the unit director and the appointment of a physician who opposes abortion (in view of her public opinion on social media).

In addition to being part of a set of actions aimed at preventing the exercise of sexual and reproductive rights, these initiatives increase the moralization and condemnation of abortion in public debates, worsening the existing inequities in access to legal and safe abortion and in health care in cases of complications due to unsafe abortions. This whole context exemplifies one of the central arguments that make up the theoretical framework on reproductive governance. As Briggs warns ^24^, “all policies are reproductive policies” since the development needs of the capitalist State are intimately related to the management of reproductive work, which includes reproduction itself, the work of caring for and feeding children and older adults, and all that makes possible the maintenance of human life. Thus, gestating bodies remain as preferential targets of State control and intervention strategies. An alliance between academia, social movements, and sectors at different levels and government levels is urgently needed to enforce the rights guaranteed by law and to defend a secular State with rights.
