# Supplementary material for: Law n. 22,537/2024 of the State of Goiás, Brazil, on awareness against abortion: a critical analysis
Source: Cad Saude Publica. 2024 Jun 21;40(6):e00016724. doi: 10.1590/0102-311XEN016724 (PMC11192571; doi:10.1590/0102-311XEN016724)
Supplement: Translation

# Translation: *Lei nº 22.537/2024*, do Estado de Goiás, Brasil, de conscientização contra o aborto: uma análise crítica

**DOI:** 10.1590/0102-311XPT016724

**Authors:** Roberta Siqueira Mocaiber Dieguez, Cristiane da Silva Cabral

**Affiliations:** 1 Faculdade de Saúde Pública, Universidade de São Paulo, São Paulo, Brasil.

No dia 11 de janeiro de 2024, foi sancionada na Assembleia Legislativa de Goiás a *Lei nº 22.537/2024* ^1^, que institui a “Campanha de Conscientização Contra o Aborto para as Mulheres no Estado de Goiás”. Proposta pelo então deputado estadual Fred Rodrigues, a lei prevê medidas de combate ao aborto no Estado de Goiás. No entanto, o documento não menciona em momento algum os permissivos legais para o aborto no Brasil: (i) risco à vida da gestante; (ii) gravidez decorrente de estupro; e (iii) anencefalia fetal. Embora tenha sido alvo de uma Ação Direta de Inconstitucionalidade (ADI 7597) no Supremo Tribunal Federal (STF), a referida lei ainda está em vigor.

Alinhado às principais estratégias adotadas recentemente por grupos que se posicionam contra o aborto ^2^ e a outros Projetos de Lei que tramitam ou tramitaram recentemente no Congresso Nacional ^3^ ^,^ ^4^ ^,^ ^5^, o argumento central do documento goiano é a proteção à vida do “nascituro”. A atribuição de subjetividade ao feto não nascido objetiva produzir um falso conflito de direitos entre a gestante e o feto que ela carrega ^6^. Trata-se de uma evidente violação dos direitos reprodutivos da pessoa que gesta.

No 1º parágrafo do art. 3º, o documento prevê o desenvolvimento de palestras, direcionadas a crianças e adolescentes, sobre os supostos “riscos” do aborto, sem distinguir entre interrupções clandestinas (em grande medida, são abortos inseguros) e aborto legal. A literatura científica recente alerta para os riscos do aborto inseguro, sobretudo em países com leis mais restritivas, como no Brasil ^7^. Não há dúvidas quanto à eficácia e segurança do aborto legal ^8^ quando utilizados os métodos recomendados ^9^.

Entretanto, um dos pontos que chamam a atenção nessa proposta é o enfoque dado às crianças e adolescentes, que são as principais vítimas de violência sexual no Brasil. As mais afetadas por esse problema são do sexo feminino (88,7%) e na faixa etária de 10 a 13 anos (33,2%) ^10^. A lei aprovada em Goiás pretende levar informações incorretas para crianças e adolescentes que possivelmente são vítimas de violência sexual e, portanto, têm direito ao aborto legal. Vale ressaltar que gravidez em menores de 14 anos significa estupro presumido e, portanto, a gestante tem direito ao aborto previsto em lei desde 1940.

Outra diretriz, presente no 2º parágrafo do art. 3º, prevê a informação da população sobre métodos contraceptivos, a fim de prevenir casos de gravidez não planejada. Embora a ampliação do acesso a informações sobre os métodos contraceptivos seja sempre algo positivo do ponto de vista da saúde pública, incluir esse tópico em uma lei contra o aborto sugere que a gravidez imprevista decorre, principalmente, da falta de acesso a informações. Entretanto, o uso eficaz da contracepção extrapola o acesso ao conhecimento sobre o assunto, na medida em que envolve uma complexa rede de relações sociais, como marcadores sociais da diferença (classe social, raça, gênero, estruturação dos serviços etc.), desigualdades nas relações afetivo-sexuais e a história individual ^11^. Sendo assim, a associação imediata entre contracepção e falta de informação reforça uma narrativa que culpabiliza somente as mulheres pela gravidez, enquanto isenta os homens e desconsidera as complexidades que atravessam a contracepção. Esse é mais um equívoco embutido na referida lei, que reforça o estigma da gravidez imprevista enquanto resultado de um fracasso das mulheres na regulação de sua fecundidade ^12^.

O tópico de maior repercussão da aprovação do texto foi o “fornecimento compulsório” de ultrassonografia para as mulheres grávidas, que passam a ser obrigadas a ouvir os batimentos cardíacos do feto (art. 3º, parágrafo 6º). Além de configurar violência institucional obstétrica, entende-se que essa medida visa impedir a intenção justamente daquelas que não desejam dar seguimento à gravidez. Como observado em outro estudo ^13^, o uso do ultrassom na atenção ao aborto confere “atributos de sujeito social” ao feto, favorecendo a condenação moral da mulher que opta pelo aborto e colocando-a em risco de denúncia.

Por fim, o texto propõe: “*estimular a iniciativa privada e ONGs na promoção de meios para acolher, orientar e prestar assistência psicológica e social às mulheres grávidas que manifestem o desejo de abortar, priorizando sempre a manutenção da vida do nascituro*” (art. 3º, parágrafo 5º). A priorização da vida do feto se sobrepõe ao direito da gestante ao aborto legal, o que induz a manutenção de uma gravidez indesejada, ainda que o direito à interrupção seja manifesto. Trata-se, assim, de uma forma de produzir impeditivos de ordem moral, psicológica e institucional para quem precisa interromper uma gravidez, aumentando o imenso número de barreiras já existentes para o aborto previsto em lei.

Barreiras e dificuldades de acesso ao aborto seguro podem resultar em diversos danos à saúde e às condições de vida das pessoas envolvidas. A manutenção da gravidez devido à negação do aborto pode levar a consequências psicológicas, sociais, econômicas, relacionais, entre outras ^14^. Na saúde, a negativa do aborto pode levar a maiores riscos de condições como eclâmpsia e hemorragia pós-parto, podendo inclusive aumentar o risco de morte materna ^15^, além de estar associada a maiores índices de depressão pós-parto ^16^. Além disso, propostas que restringem o acesso ao aborto legal e seguro contrariam as mais recentes recomendações da Organização Mundial da Saúde ^8^, para as quais leis menos restritivas para o aborto são um importante passo para a promoção da saúde e dos direitos sexuais e reprodutivos de mulheres e homens.

Portanto, a aprovação da *Lei nº 22.537/2024* tem como objetivo o impedimento do acesso ao aborto legal e como consequência a imposição da gravidez em casos de estupro, violando o Código Penal brasileiro que confere o direito ao aborto nesses casos. Ainda que não seja possível que a lei federal seja alterada por uma lei estadual, a proposta impõe sofrimento psicológico desnecessário à gestante ao tentar forçar a continuidade da gravidez decorrente de estupro e agrava as já conhecidas barreiras de acesso relacionadas à objeção de consciência, ao limite de tempo gestacional, à distribuição dos serviços ^17^ ^,^ ^18^ ^,^ ^19^, entre outras.

Embora a mudança só tenha impacto direto no Estado de Goiás, consideramos que sua aprovação contribui para fortalecer projetos de lei semelhantes em tramitação em outros estados da Federação. Essas mudanças na legislação são fortes mecanismos institucionais que interferem na autonomia e gestão reprodutiva (não apenas de pessoas que engravidam) e contribuem para a produção de regimes morais ^20^ ^,^ ^21^ ^,^ ^22^ que atuam de forma coercitiva sobre os diferentes sujeitos, definindo modos de viver a vida. Atualmente, existem vários projetos de lei no país que se alinham a uma agenda neoconservadora e antiaborto, propostos por representantes do legislativo municipal, estadual ou federal. Somam-se a isso outros ataques recentes aos direitos sexuais e reprodutivos, que se manifestam, por exemplo, no fechamento de serviços de referência para atenção ao aborto legal. Um exemplo é o Hospital Vila Nova Cachoeirinha, na cidade de São Paulo, considerado um dos únicos do Brasil a oferecer o procedimento em casos de tempo gestacional superior a 22 semanas. O prefeito da cidade determinou a interrupção do serviço de aborto legal em dezembro de 2023, sob alegação de priorizar outros procedimentos e cirurgias ^23^. Na sequência, houve a demissão do diretor da unidade e a nomeação de uma médica sabidamente contrária ao aborto, tendo em vista seus posicionamentos públicos em redes sociais.

Além de integrarem um conjunto de ações que visam impedir o exercício dos direitos sexuais e reprodutivos, essas iniciativas têm como consequência o aumento da moralização e da condenação do aborto nos debates públicos, o que agrava as já conhecidas iniquidades no acesso ao aborto legal e seguro e na atenção à saúde nos casos de complicações causadas pelo aborto inseguro. Todo esse contexto é exemplar de um dos argumentos centrais que compõem o arcabouço teórico sobre governança reprodutiva. Conforme alerta Briggs ^24^, “*todas as políticas são políticas reprodutivas*”, já que as necessidades de desenvolvimento do Estado capitalista se relacionam intimamente com a gestão do trabalho reprodutivo, o que inclui a reprodução em si, o trabalho do cuidado e da alimentação de crianças e idosos e todo o trabalho que possibilita a continuidade da vida humana. Assim, os corpos que gestam permanecem como alvos preferenciais das estratégias de controle e de intervenção do Estado. Urge uma aliança entre academia, movimentos sociais e setores de diversos níveis e instâncias governamentais que cumpra os direitos já assegurados em lei e defenda um Estado laico e de direitos.
